# The Role of Ketogenic Metabolic Therapy on the Brain in Serious Mental Illness: A Review

**DOI:** 10.20900/jpbs.20220009

**Published:** 2022-10-31

**Authors:** Shebani Sethi, Judith M. Ford

**Affiliations:** 1 Metabolic Psychiatry, Stanford University School of Medicine, Stanford, CA 94305, USA; 2 Mental Health, San Francisco Veterans Affairs Medical Center, San Francisco, CA 94121, USA; 3 Department of Psychiatry and Behavioral Sciences, University of California, San Francisco, CA 94121, USA

**Keywords:** schizophrenia, bipolar disorder, insulin resistance, metabolism, functional connectivity, neural network stability, psychotic symptoms, metabolic psychiatry

## Abstract

In search of interventions targeting brain dysfunction and underlying cognitive impairment in schizophrenia, we look at the brain and beyond to the potential role of dysfunctional systemic metabolism on neural network instability and insulin resistance in serious mental illness. We note that disrupted insulin and cerebral glucose metabolism are seen even in medication-naïve first-episode schizophrenia, suggesting that people with schizophrenia are at risk for Type 2 diabetes and cardiovascular disease, resulting in a shortened life span. Although glucose is the brain’s default fuel, ketones are a more efficient fuel for the brain. We highlight evidence that a ketogenic diet can improve both the metabolic and neural stability profiles. Specifically, a ketogenic diet improves mitochondrial metabolism, neurotransmitter function, oxidative stress/inflammation, while also increasing neural network stability and cognitive function. To reverse the neurodegenerative process, increasing the brain’s access to ketone bodies may be needed. We describe evidence that metabolic, neuroprotective, and neurochemical benefits of a ketogenic diet potentially provide symptomatic relief to people with schizophrenia while also improving their cardiovascular or metabolic health. We review evidence for KD side effects and note that although high in fat it improves various cardiovascular and metabolic risk markers in overweight/obese individuals. We conclude by calling for controlled clinical trials to confirm or refute the findings from anecdotal and case reports to address the potential beneficial effects of the ketogenic diet in people with serious mental illness.

## INTRODUCTION

Traditionally, nutrition has been used as adjunctive therapy for improving lipid profiles, blood glucose, insulin resistance, and diabetes, however, it has not been thought of as a metabolic therapy affecting the structure and function of the brain, despite preliminary evidence otherwise [1]. For example, diet has been shown to have an effect on core symptoms of pediatric epilepsy [2]. Recent therapeutic focus has shifted towards the influence of nutrition on neural network brain stability, brain-derived neurotrophic factor, ATP energy function and neurotransmitter balance [1,3]. Diet, in particular ketogenic diets, have been identified to influence several biological processes, including mitochondrial energy metabolism, inflammatory processes, oxidative stress, monoaminergic activity, and progression of neuro-degeneration, and hence are considered a metabolic therapy itself [4]. Many neurological diseases, such as Alzheimer’s disease (AD), Parkinson’s disease (PD), epilepsy, bipolar disorder (BD), schizophrenia (SZ), and major depressive disorder (MDD) are characterized by cerebral glucose hypometabolism, insulin resistance, neurotransmitter imbalances, mitochondrial dysfunction, oxidative stress, and inflammation as potential causative factors [5,6]. Insulin resistance is a risk factor for dementia [7], cognitive deterioration later in life in those with type 2 diabetes mellitus (T2DM), and mood disorder, such as depression [8,9] as well as cognitive dysfunction in youth [10]. Reductions in left hippocampal grey matter volume have also been found to be common to MDD, BD, and SZ [11], showcasing the close neural interaction shared by these conditions. Therefore, new interventional approaches of metabolic psychiatry prevention and treatment targets must be further studied and may have the potential to yield universal improvements in psychiatric conditions through neuronal access to metabolic changes with nutritional ketones [11]. We review the current body of evidence for the effects of Ketogenic Diets (KD) on neuronal networks.

**Figure 1.**
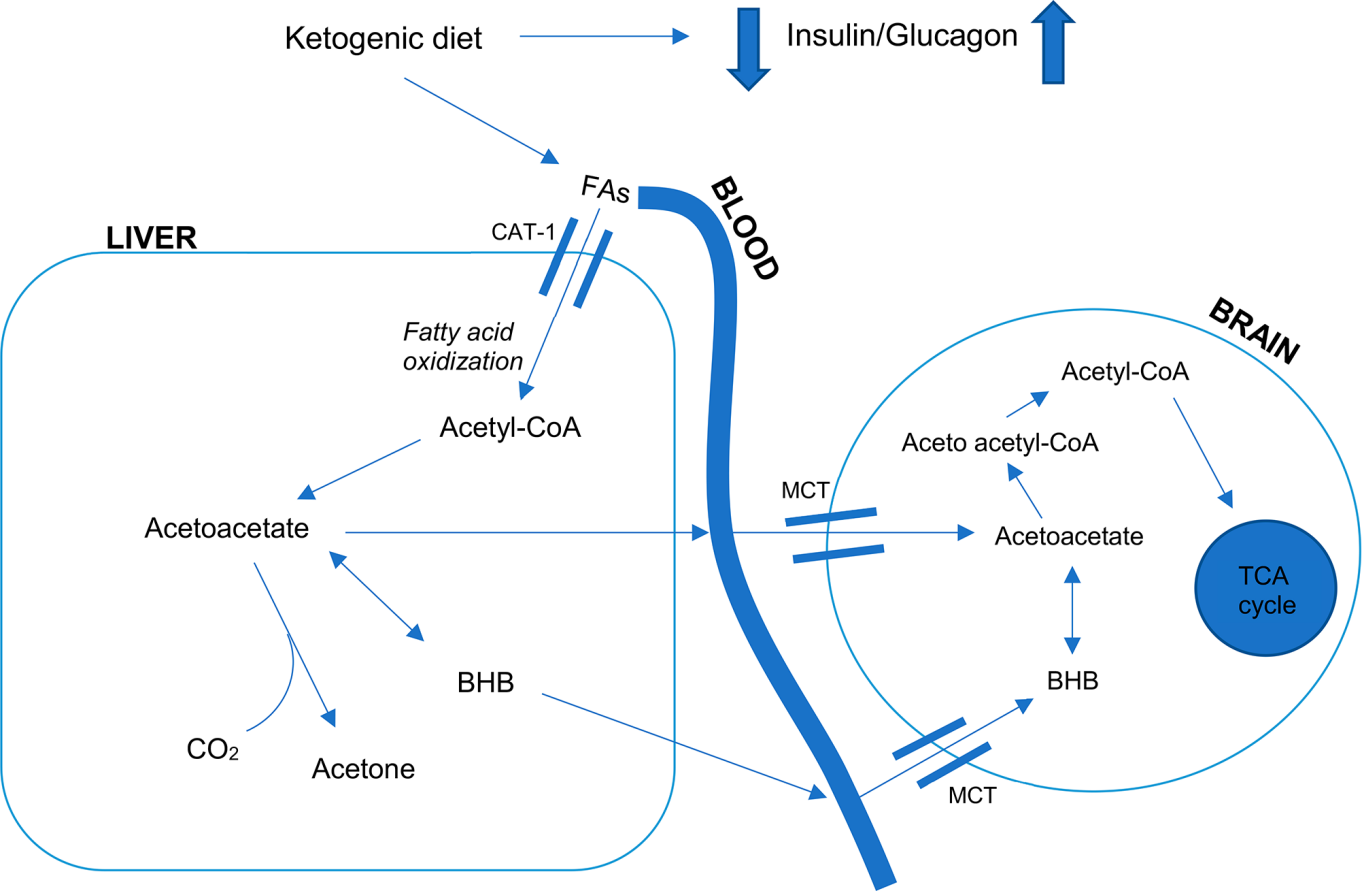
A depiction of the biochemistry of ketogenesis in the liver and brain. Prolonged glucose restriction leads to an increased glucagon to insulin ratio, which leads to release of free fatty acids into the bloodstream. Free fatty acids are taken up into liver mitochondria where they are used to produce acetyl coenzyme A (Acetyl-CoA). These molecules then enter ketogenesis through the formation of ketone bodies. Acetyl-CoA is converted into acetoacetate, which then allows for reversible reduction to beta hydroxybutyrate (BHB), as well as acetone. These ketone bodies then exit the liver and enter peripheral tissues and the brain, which is facilitated by monocarboxylic acid transporters. When in situ, BHB can be converted back into acetoacetate, serving as an eventual source of acetyl-CoA to release energy via the tricarboxylic acid cycle. Abbreviations: Acetyl-CoA, acetyl coenzyme A; BHB, beta- hydroxybutyrate; CAT, carnitine acylcarnitine translocase; CO_2_, carbon dioxide; FAs, fatty acids; MCT, monocarboxylic acid transporter; TCA, tricarboxylic acid.

The KD has been identified as a potential treatment for neurodegenerative and neuropsychiatric conditions [12–14]. Initially used by clinicians in the 1920s as a treatment for epilepsy, this high-fat, moderate protein, low-carbohydrate diet releases ketone bodies (principally β–hydroxybutyrate (β-HB) and acetate) from the breakdown of fat and serves as an alternative fuel, diverting away from the use of glucose as the body’s main energy source [15]. See figure 1. Adhering to a sustained KD, an individual achieves a level of nutritional ketosis, contrary to and well below pathological ketoacidosis by diet instead of starvation. [16]. During times of glucose deprivation or increased energetic demands, the brain has evolved to utilize ketones to preserve and augment critical central functions [17]. This is evident in a fasting state such as during sleep, when ketones can increase and maintain circulating ketone bodies, especially β-HB. Increased levels of β-HB have been reported to improve symptoms of various age-related diseases [18], thereby providing a rationale for the development of therapeutic ketogenic interventions in neurodegenerative diseases [19].

## KETONES ARE FUEL FOR THE BRAIN AND BODY

Although the human brain is only 2% of the body’s volume, it consumes over 20% of its energy at rest [20], and accordingly, the brain is particularly vulnerable to changes in metabolism. While glucose is normally considered to be the brain’s default fuel, ketones provide 27% more free energy than glucose [21]. People with insulin resistance cannot use glucose effectivity, with obvious consequences for brain function such that insulin resistance is an early risk-factor for dementia later in life [9]. In neurodegenerative conditions, the brain is unable to use glucose effectively due to both glial and neuronal changes in glucose transportation, in addition to changes in cellular respiration enzymatic activities, and insulin signaling [17]. During times of glucose deprivation or increased energetic demands, the brain has evolved to utilize ketones to preserve and augment critical central functions thereby providing a rationale for the development of therapeutic ketogenic interventions in AD and other neurodegenerative diseases. Ketones are released from free fatty acids taken up by the liver after glycogen stores are depleted in a fasting state. Mattson et al. suggest that this fuel switch is accompanied by biological adaptations of neural networks in the brain that optimize their function [14]. As might be expected, cognitive impairments in schizophrenia are related to brain insulin resistance, supporting its role in the pathophysiology of cognitive dysfunction in SZ [22]. Ketones are anti-inflammatory, decrease production of reactive oxygen species, and upregulate mitochondrial biogenesis in the brain [16].

## BENEFITS OF THE KETOGENIC DIET

**Figure 2.**
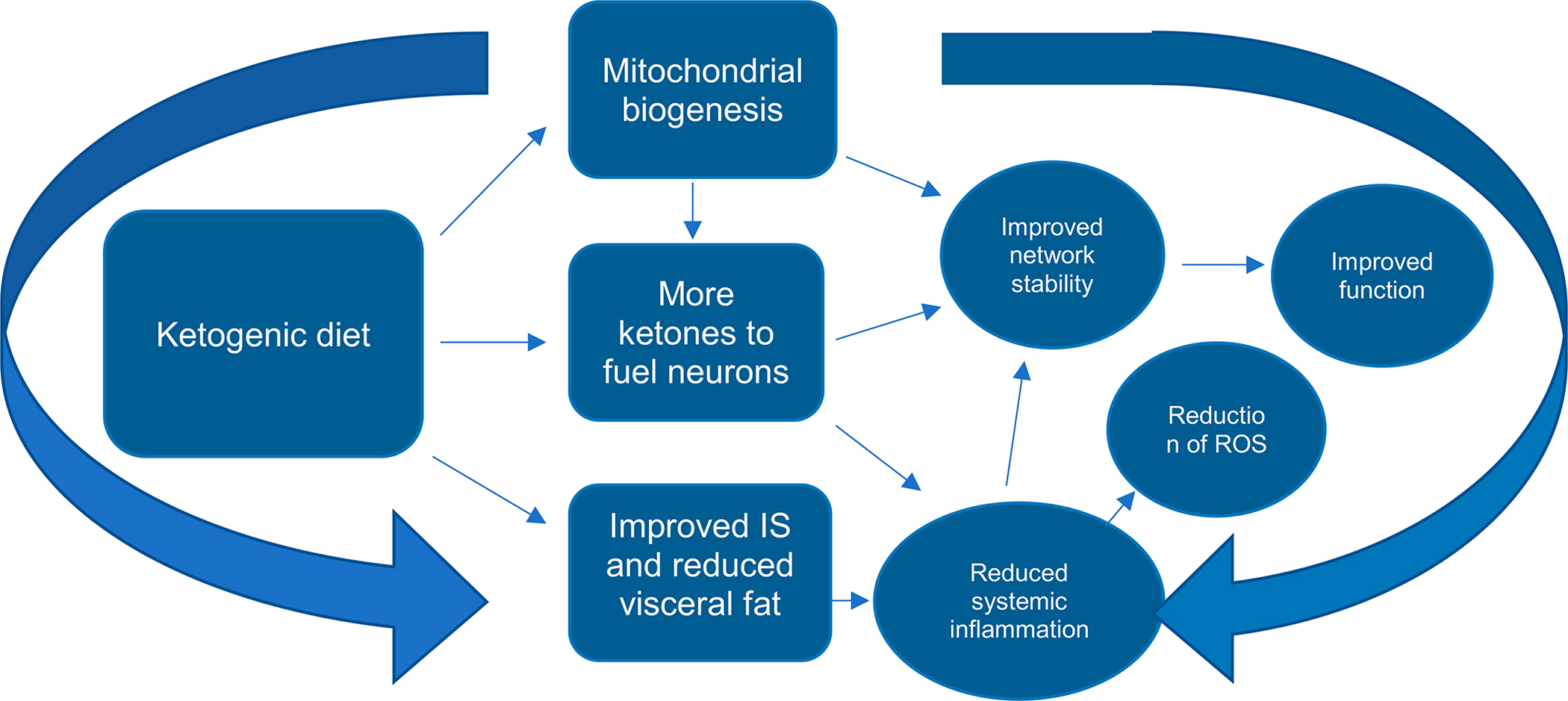
A diagram depicting a basic mechanistic model of the ketogenic diet and its potential benefits. Neurobiological and physiological mechanisms of the ketogenic diet are shown in rectangular boxes, with corresponding effects in circles. The flow chart depicts at a high level possible mechanisms of ketogenic diet on cognition and mental health functioning. Abbreviations: IS, insulin sensitivity, ROS, reactive oxygen species.

Nutritional ketosis is associated with improvement in metabolic health and mitochondrial function [16]. For example, a randomized controlled trial of 119 participants by McClernon et al. [23] reported participants assigned to a KD versus a low fat diet had significant decreases in body mass index (BMI) after six months, alongside mood improvements, and a significant reduction in negative affect and hunger [20]. Similarly, participants in an uncontrolled intervention study experienced a decrease in insulin levels and BMI, as well as an improvement in cognitive function assessing working memory and speed of processing after 12 weeks [24]. As a result of the extracellular changes that occur during ketosis, intracellular sodium concentrations would be expected to decrease correspondingly, which is a common feature of mood-stabilizing medications [25]. The utilization of ketone bodies by the brain instead of glucose has been proposed to bypass glucose hypometabolism commonly associated with neurological diseases, evidenced in a study by Cunnane et al. [26] who found that uptake of ketone bodies in individuals with AD has a beneficial effect on cognitive outcomes. Ketone bodies may also provide neural benefits to younger individuals and those not yet in a hypometabolic state, as ketones increase Gibbs free energy exchange for ATP by 27% compared to glucose, potentially representing a more efficient fuel for the brain [21,27]. In addition to bypassing glucose hypometabolism in the brain, ketone bodies have several favorable metabolic adaptations in regards to neurotransmitter imbalances, oxidative stress, and inflammation, characteristic of several neurological diseases [5]. While there may be other neurobiological mechanisms, see Table 1 and Figure 2 for potential mechanistic effects.

Imbalance of the GABA/glutamate neurotransmitters and glutamate excitotoxicity are predominant features of neurological diseases, from epilepsy [28] to AD [29], which have been shown to be corrected by KD [29–31]. A study by Olson et al. 31 demonstrated that a KD reduced seizures in a mouse model of epilepsy and that this was associated with an increase in GABA/glutamate and decrease in excitotoxicity. Similarly, in another study, Kraeuter et al. [32] pharmacologically manipulated GABA/glutamate balance to generate a mouse model of SZ and reported normalization of symptoms after three weeks of exogenous β-HB administration. It has been generally accepted that oxidative stress contributes to most, if not all, chronic diseases, including SZ, BD, and MDD [6]. The KD has a myriad of corrective mechanisms of oxidative stress in neurological disorders, which have been reviewed in depth elsewhere [30,33]. Oxidative stress and inflammation are mutually reinforcing disease states [5,30], with recent post-mortem and in-vivo human evidence demonstrating the association between brain inflammation and mental illness [34]. This is also seen in other mental illnesses, as a study by Marques et al. (2019) found increased inflammatory markers (translocator protein) in the brains of living SZ patients [35].

A 2019 study by Athinarayanan et al. [36] investigated the effects of the KD compared to usual care in patients with T2DM over two years, finding significant improvements in restoring cardiometabolic function whilst utilizing less medication. This was evident through reductions in HbA1c, fasting glucose, fasting insulin, BMI, blood pressure, and triglycerides in the KD group. There was also a resolution of diabetes in the KD group (53.5% reversal, 17.6% remission) but not in the control group. Similar reductions in HbA1c, BMI, and medication use when comparing KD to usual care in T2DM patients have been reported in other studies investigating effects after 10 weeks and one-year [37,38]. Furthermore, a recent five-year clinical trial of the KD in patients with T2DM has found similar positive cardiometabolic changes, demonstrating the potential for beneficial long-term outcomes [39]. The increase of small LDL particles is a common characteristic of diabetic dyslipidemia, and this has been found to be reversed by a KD [40]. Correspondingly, these positive cardiometabolic changes have been credited to lower the risk of cardiovascular disease in the T2DM population. Conversely, a recent review by Parry-Strong et al. [41] investigated the effects of the KD on T2DM, concluding that the diet may cause reductions in HbA1c, however, evidence of an advantage over other strategies is limited and further research is needed to provide definitive evidence.

## CLINICAL EVIDENCE OF THE KETOGENIC DIET IN NEUROLOGICAL CONDITIONS

The KD first came to prevalence following its use in epilepsy in the 1920s and is currently mainly used in children with treatment resistant seizures [42]. Current research investigating the KD in epileptic adults does not show effects as favorable to those found in children, with fewer adult studies reporting seizure freedom or reduction compared to studies in children [42,43], possibly because adults typically fix their own meals and their eating is not monitored. A 2018 randomized controlled trial by Kverneland et al. [44] investigated the effects of a modified Atkins diet on adult epileptic patients. This diet also induces ketosis by limiting individuals to a maximum carbohydrate intake of 20 g/day. When compared to a control group, the intervention group showed significant reductions in seizure frequency, however, this was only a moderate reduction of 25%.

The accumulation of amyloid plaques through mitochondrial dysfunction, glucose hypometabolism, and neuronal loss are hallmark features of AD [29,45]. Recent management strategies for AD have been aimed at modifying dietary and lifestyle habits, with the KD gaining traction as an intervention [29]. Several preclinical studies on the KD in Alzheimer’s have yielded promising results. Circulating ketone bodies of β-HB were found to attenuate the toxic effects of the amyloid beta peptide and protect mitochondrial function [46]. Additionally, studies in animal models have proved encouraging, with Van Der Auwera et al. [47] finding a 25% reduction in amyloid beta levels in mice on a KD compared to controls.

The use of the KD as an anticonvulsant intervention in BD was first proposed in 2001 by El-Mallakh and Paskitti [25], highlighting the diet’s positive effects on glucose hypometabolism. However, there has been a lack of available human data investigating KD in BD. The first case series by Phelps, Siemers, and El-Mallakh [48] focused on two female patients with BD who were assigned a KD and maintained nutritional ketosis for up to three years. Both patients experienced the mood stabilizing effects commonly seen with medication, with no adverse reactions reported. It was hypothesized that the diet reduced intracellular sodium and calcium, which acidified blood plasma and stabilized mood [48]. The energy metabolism of ATP generated in BD is incapable of sustaining the sodium-potassium pump in neurons, which may cause a depressed state in conditions of severe ATP use deficiency, and a manic state in less severe ATP use deficiency [49]. This has led to the KD being hypothesized as an effective therapeutic intervention in BD due to positive effects on mitochondrial metabolism and function [50]. The underlying characteristics shared by these neurological conditions can also be evidenced in MDD. The mood stabilization effects of the KD have been identified as potentially recreating the pharmacological effects of mood stabilizing medication whilst circumventing detrimental side effects [15,25]. Reductions in neuroinflammation associated with KDs has been suggested to provide antidepressant effects and subsequently improve symptoms in patients with mood disorders [51,52]. Similarly in PD, the antioxidant and anti-inflammatory effects of the KD have been identified as neuroprotective mechanisms to potentially slow or halt progression of the disease [33]. Research has found that the presence of β-HB in PD patients have been found to be neuroprotective, supporting KD as a therapeutic intervention for PD [46,53].

Effective glucose metabolism maintains global excitatory neural network function [54]. Therefore, low availability of energy substrates can reduce synaptic function and lead to neural network instability [55], as is the case with the glucose hypometabolism that the discussed neurological conditions share. Neural network instability has recently been identified as a potential link to recurrent seizures in epilepsy and the use of the KD as a metabolic therapy has been speculated to provide a buffer against neural excitability and promote normal function [56]. The effects of the KD on mitochondrial function may be from improving ATP energy metabolism, likely improving neuronal homeostasis and also enabling higher resilience to neural damage during seizures [57]. The link between epilepsy and SZ has been well established, and the efficacy of some anti-epileptic medications in SZ patients suggests shared disease mechanisms [58]. There seems to be an association between metabolism and neural network stability and given the established success of the KD as a therapeutic intervention in epilepsy, it is likely that it will produce the same results in SZ.

## SCHIZOPHRENIA, METABOLISM, INFLAMMATION, AND NEURONAL TARGETS

SZ is diagnosed based on positive symptoms, such as delusions and hallucinations, and negative symptoms, such as anhedonia and amotivation. It is also characterized by cognitive deficits that are responsible for poor social and occupational outcomes [59]. Neuroleptic medications treat positive symptoms, but they have not been able to improve cognition, nor do they target pathophysiological mechanisms thought to underlie these deficits. Furthermore, antipsychotic use is frequently associated with motor and metabolic side effects [54]. Therefore, research is additionally focusing on both interventional strategies targeting brain dysfunction and the potential role of systemic metabolic dysfunction. In understanding the etiology of SZ, a leading theory is the abnormal neurodevelopment hypothesis which includes the influences of genetics, prenatal and perinatal disorders, and its combined interaction with environmental factors [60]. Another condition characterized by abnormal neurodevelopment is epilepsy [61], and given the observed ability of the KD to improve symptoms of epilepsy in pharmaco-resistant children [42], it suggests the possibility that the KD may be beneficial in controlling other potential neurodevelopmental conditions such as SZ.

As mentioned, insulin resistance and obesity have been historically linked to SZ, even before the advent of antipsychotic medication [62]. Importantly, antipsychotic medication may worsen cognitive dysfunction in SZ patients [63]. A recent meta-analysis affirms the presence of disrupted glucose metabolism and insulin resistance in medication-naïve first-episode SZ patients, suggesting that SZ itself, and not just the medication used to treat it, increases the risk of T2DM, cardiovascular morbidity and mortality, and more generally, accelerated aging [62]. Even young people with SZ are prone to diseases associated with aging including metabolic disease [64,65] and cognitive deficits [66,67]. Mitochondrial dysfunction is a potential mechanism underlying the association between SZ and glucose dysregulation [68]. SZ is also associated with systemic inflammation, as a study found significantly increased inflammatory markers on PET scan in the microglia of SZ patients compared to healthy controls [35]. Genome-wide studies have confirmed patients with SZ to have an inherent genetic predisposition to insulin resistance [69,70].

## FUNCTIONAL DYSCONNECTIVITY IN SCHIZOPHRENIA

Human neuroscience has benefitted from the use of functional MRI (fMRI) to elucidate the functional neuroanatomical underpinnings of cognition associated with injury, illness, and age. fMRI has been used for more than two decades to assess neural function in specific regions of the brain as subjects perform a variety of tasks varying in difficulty and made more difficult by the overlay of scanner noise and physical restraint. More recently, the field discovered that much can be learned about the function of the brain by studying spontaneous oscillations of brain activity during rest, avoiding confounding factors of motivation and intellect for task performance [71]. Just as significant was the discovery that functional connectivity could be assessed by correlating oscillating activity in one region of the brain with another. This likely reflects the scaffolding between different brain areas, when they are repeatedly co-active, recalling Hebb’s rule: “units that fire together, wire together” [72]. While functional dysconnectivity is related to cognitive deficits in SZ, it is not specific to a particular neural network or cognitive domain [73]. As might be expected, this resting-state functional connectivity is consistent with structural connectivity, as it is calculated over long periods of time [74].

Accordingly, most of the functional connectivity literature reports on static measures of connectivity, without regard to state fluctuation or transitions between moments in time in the resting scan time series. Recently, we [75–78] and others [1] have broadened this to include measures of functional network stability from moment to moment. Specifically, network stability reflects dynamic connectivity by assessing how long a network of independent nodes, within and between brain regions, maintains a stable connection. Network instability increases with age, cognitive deficits, and in T2DM [1].

## KETOSIS STABILIZES BRAIN NETWORKS

Muiica-Parodi et al. [1] reported that a one-week KD increases functional brain network stability, restoring it to that seen in younger people. They showed that in younger (<50 years old) adults, nutritional ketosis stabilized functional networks. Most importantly, in a separate, larger sample, they found network instability increased with age and with decreases in cognitive functioning [1], with the aging effect being accelerated in young people with T2DM. Although ketosis has a significant cumulative and synergistic effect over the years, these network changes occurred with a single week of ketosis, suggesting short-term adaptations to network stability are feasible with a KD. Ruling out any effects of weight loss on network stability, the authors reported similar network stabilization when giving participants a single exogenous ketone ester drink.

## KETOGENIC DIETS AND SYMPTOMS IN SCHIZOPHRENIA

It is thought that the mechanism of KD bypassing glucose hypometabolism in patients with SZ helps increase oxygen consumption, improves ATP energy metabolism, and induces brain-derived neurotrophic factor to improve cognition [3,54,79]. In a postmortem analysis study by Sullivan et al. [80] investigating the brains of mouse models of SZ, the authors reported a 19%–22% decrease in glucose transporter expression, GLUT1 and GLUT3, and in glycolytic genes. These brains also unveiled a 22% increase in the β-HB importer (MCT1), suggesting that the brain may be compensating for cerebral glucose hypometabolism by upregulating its facility to transport ketone bodies. Therefore, the brain with SZ may be metabolically prepared to respond to a KD. Further studies of KD in animal models have yielded favorable results [32,81,82], however, clinical evidence in human subjects is limited to case reports and small pilot studies [83–85]. A case report by Palmer [85] reported on two instances of SZ patients who experienced a drastic improvement in symptoms after adopting a KD. Neither patient started a KD to treat their SZ, however, within two-to-four weeks, both patients noticed a dramatic reduction in symptoms of psychosis and subsequently stopped all antipsychotic medications. Similar results were reported in a case study by Kraft and Westman [83], whereby a patient with a 50-year history of SZ reported a resolution in longstanding symptoms of auditory hallucinations after one week of initiating a KD. Upon 12-month follow-up, the authors reported that the patient was able to adhere to the diet, only having 2–3 isolated episodes of consuming carbohydrates around holidays, however, these periods did not correspond with a recurrence in her symptoms.

A pilot case series by Gilbert-Jaramillo et al. [86] investigated the effects of a 2000 kcal, 3:1 KD (3 parts fat to every 1 part protein and carbohydrate) over six weeks on twins diagnosed with SZ. Both participants had tried numerous medications to resolve their symptoms, however, these were unsuccessful. Medications were continued throughout the study. Unfortunately, both participants struggled with compliance to the diet, reporting difficulty due to onset of severe high sugar food cravings after 14 days of the KD. The Positive and Negative Syndrome Scale (PANSS) was used as a measure of SZ symptoms, which decreased modestly alongside body fat over the six-week intervention. Although the study showed that the KD can have short-term benefits on psychiatric condition, metabolic function and body composition in young adults, results were limited due to a lack of compliance to the KD. Efforts to improve the compliance are needed for the field to move forward, and alternate ways to promote ketosis should be explored. Ultimately, blood ketone levels should be monitored to allow the most flexibility in ketogenic treatments.

A recent study by Danan et al. [87] investigated the effects of the KD on patients with severe, persistent mental illness whose symptoms were poorly controlled with neuroleptic medication. Of the 31 patients, 12 were diagnosed with SZ, however, two of the SZ patients dropped out due to inability to adhere to the KD for >14 days. Throughout the duration of the study period, the patients were voluntarily admitted to a psychiatric hospital 6 days per week to allow for close monitoring. During these periods they were given ketogenic meals, however, for up to 36 consecutive hours on the weekends they were unsupervised. The duration of the intervention ranged from 6 to 248 days, with significant improvements in symptoms of depression (Hamilton Depression Rating Scale, Montgomery-Åsberg Depression Rating Scale) and SZ (PANSS), alongside metabolic health measures of BMI, blood pressure, blood glucose, and triglycerides. All 10 patients with SZ recorded improvements in PANSS scores, with a mean reduction from 91.4 to 49.3. The minimal clinically significant change in PANSS of 16.5 was achieved in all 10 patients, however, the average reduction of 42.1 points is far above this and is therefore supportive of the KD as an interventional strategy for SZ [88]. Study limitations include retrospective data, sample, and unique controlled conditions where intervention was applied. Also, there was no hospitalized, diet as usual control group for comparison with the KD patients; it is possible that just being in the hospital is associated with improvement in PANSS. The high compliance rate of 90% was likely due to food being prepared 6 days per week in a controlled monitored setting.

Preliminary analytic data of approximately half (13) of the participants to date in a Stanford open label, single arm pilot trial in an outpatient population was recently presented and revealed benefits with the KD on patients with BD and schizophrenia. This cohort included 13 patients, 10 with BD and 3 with schizophrenia, with 1 drop out. Participants were provided KD metabolic therapy for 16 weeks and had initially weekly and after one-month, biweekly clinical evaluations with a psychiatrist and nutritionist coach. Metabolic improvements achieved overall included 10% decrease in BMI, 19% reduction in absolute fat mass, systolic/diastolic blood pressure decrease, and 31% reduction in visceral adipose tissue. Metabolic syndrome was reversed in all who met criteria at the outset of the study (3). Additional metabolic biomarkers improvements included a 28% decrease in hs-CRP, a high sensitivity inflammatory metabolic marker and 21% reduction in triglycerides. Psychiatric improvements were also observed, with an overall 16% improvement in life satisfaction (MANSA Quality of Life), 34% improvement in Clinical Global Impression, 25% reduction in depressive symptoms on patient health questionnaire and 28% reduction in sleep quality with Pittsburgh Sleep Quality Index [15,89]. The preliminary results suggests that a KD as a metabolic and mental clinical therapeutic intervention offers promise.

## SAFETY AND COMMON SIDE EFFECTS OF KETOGENIC THERAPIES

Among 16 published controlled clinical trials with more than 25 subjects for parallel design, or fewer than 15 subjects for crossover design, total cholesterol decreased in one study [90], increased in one study [53], and did not change in other 6 studies [91–96]. High-density lipoprotein cholesterol increased in 4 out of 12. Low-density lipoprotein cholesterol was unchanged in most studies, but increased in two studies [53,97]. Triglycerides decreased by 50 percent in reported studies [91,94,95,97–100] and blood pressure decreased by 33 percent [96,97]. C-reactive protein significantly decreased in one study [97]. These data suggest a KD improves various cardiovascular risk markers in overweight/obese subjects.

The adverse effects most commonly reported initially in KDs include fatigue, constipation, weight loss, and transient hyperlipidemia [14,25], however, these side effects have been found to improve with continued adherence to the diet [14]. The weight loss effect is welcome for many, particularly in individuals with obesity, however, would need to be monitored regularly depending on the medical condition. Additionally, lipid profiles in individuals starting a KD have been shown to acutely increase when beginning the diet, but normalize after approximately one year [101]. Normal healthy lipid profiles have been found to persist in long term KD use, in excess of three years [102]. It is worth noting that the carbohydrate composition of diets in studies varied, from the traditional KD which typically consists of 20 g/day, to those which consist of 50 g/day or roughly 30–40% of caloric intake. Therefore, adverse effects may not be homogenous across all studies. Individuals undergoing the medicalized version of the KD should be monitored and given corresponding supplementation if needed [14].

Diet adherence and compliance has been mixed and remains a barrier to successful application of the KD [42]. A meta-analysis of compliance rates in adults with epilepsy on the KD reported a 45% overall compliance rate [103], with the modified Atkins diet yielding higher compliance rates. Similar results were found in an observational study of 139 adult patients with epilepsy treated with a KD, 48% of patients discontinued the diet or were lost to follow-up [104]. The main reason cited for discontinuation was difficulty adhering and having enough external food choices. However, recently the food environment has shifted to become more ketogenic friendly than previously [40]. A 2018 by Hallberg et al. reported a 83% compliance rate to the KD after one year in patients with T2DM [37]. Compliance rates of other diets are not dissimilar from those previously reported of the KD, as adherence to a gluten free diet has been reported to be between 17–45% in adults with coeliac disease [105], and a 26.4% adherence to a Mediterranean diet in individuals 65 or older [106]. Recent trials of the KD in T2DM have shown adherence rates of nearly 50% at five years, whilst maintaining improvements in cardiometabolic health markers [39] and exhibiting no major adverse effects [41].

## CONCLUSIONS

In the search for interventions addressing brain dysfunction underlying cognitive impairment in SZ and bipolar illness, we look comprehensively at the brain and beyond to the potential role of dysfunctional central and systemic metabolism. Evaluating metabolic dysfunction can also help us understand the pathophysiology of serious mental illness. Diverting attention towards cardiovascular metabolism and addressing neural network stability and insulin resistance may advance developments in treatment. The mechanisms of action of a KD include efficient energy mitochondrial metabolism, neurotransmitter function, improving neural network stability and improvements in oxidative stress and inflammation. The metabolic, neuroprotective, and neurochemical benefits of the KD have the potential to provide symptomatic relief to patients, in SMI, yet this is limited by a lack of robust clinical trial data specifically in mental health. To reverse this neurodegenerative process, increasing neurons’ access to ketone bodies may be critical. Numerous clinical reviews have called for further research to confirm anecdotal and case findings [5,6,81,82], as early evidence of positive effects of the KD on schizophrenic and bipolar symptoms warrant further investigation and require confirmation through controlled clinical trials.

## Figures and Tables

**Table 1.** Potential mechanistic effects of the ketogenic diet underpinning neurological conditions.

Neural Deficit	Neural Symptom	Ketogenic Therapy Effect
Mitochondrial dysfunction	Decrease in energy level production	Induces mitochondrial biogenesis
Oxidative stress and inflammation	Increase in ROS leading to neuronal damage	Decreases ROS levels with ketone bodies; increases HDL cholesterol levels for neuroprotection
Na/K ATPase loss of function	Impaired ATP production via oxidative phosphorylation	Provides alternative energy source via ketosis, replenishes acetyl-CoA
Imbalance in monoaminergic activity	Changes in behavior and emotion due to imbalance in neurotransmitter concentrations	Regulates neurotransmitter metabolites via ketone bodies and intermediates
GABA/glutamate imbalance	Depressive and mania symptoms, unsustainable energy requirements, and neuronal damage	Increases GABA levels whilst decreasing glutamate levels

Abbreviations: ATP, adenosine triphosphate; GABA, gamma-aminobutyric acid; HDL, high-density lipoprotein; K, potassium; Na, sodium; ROS, reactive oxygen species.

## Data Availability

Data sharing not applicable to this article as no datasets were generated or analyzed during the current study.
